# Weighted Gene Co-expression Network Analysis Revealed That CircMARK3 Is a Potential CircRNA Affects Fat Deposition in Buffalo

**DOI:** 10.3389/fvets.2022.946447

**Published:** 2022-07-07

**Authors:** Xue Feng, Jinhui Zhao, Fen Li, Bandar Hamad Aloufi, Ahmed Mohajja Alshammari, Yun Ma

**Affiliations:** ^1^Key Laboratory of Ruminant Molecular and Cellular Breeding of Ningxia Hui Autonomous Region, School of Agriculture, Ningxia University, Yinchuan, China; ^2^College of Life Sciences, Xinyang Normal University, Xinyang, China; ^3^Department of Biology, College of Science, University of Hail, Hail, Saudi Arabia

**Keywords:** buffalo, circRNA, WGCNA, adipocytes, adipogenesis

## Abstract

**Background:**

Buffalo meat is increasingly widely accepted for consumption as it shares several quality attributes with cattle meat (beef). Hence, there is a huge opportunity for growth in the buffalo meat industry. However, buffalo meat has relatively low intramuscular fat (IMF) content, affecting its flavor, tenderness and juiciness. As there is a dearth of information on factors that control fat deposition, this study was undertaken to provide new candidate factor associated with buffalo fat deposition. Circular RNA (circRNA) is a novel class of non-coding RNA with a closed-loop structure, and play an important role in fat deposition.

**Methods:**

In this study, weighted gene co-expression network analysis (WGCNA) was used to construct a circRNA co-expression network and revealed a candidate circRNA that may affect the IMF deposition of buffalo as determined by RT-qPCR, semiquantitative PCR and gain-of-function experiments.

**Results:**

Herein, WGCNA determined that one module (turquoise module) is significantly associated with the growth and development stages of buffalo. Further analysis revealed a total of 191 overlapping circRNAs among differentially expressed (DE) circRNAs and the co-expression module. A candidate circRNA was found, 21:6969877|69753491 (circRNA_ID), with a reported involvement in lipid metabolism. This circRNA is stably expressed and originates from the *MARK*3 gene, hence the name circMARK3. circMARK3 is highly expressed in adipose tissue and mature adipocytes and is located in the cytoplasm. Gain-of-function experiments demonstrated that circMARK3 promoted adipogenic differentiation of buffalo adipocytes and 3T3-L1 cells by up-regulating the expression levels of adipogenic marker genes *PPARG, C/EBP*α and *FABP*4.

**Conclusion:**

These results indicate that circMARK3 is a potential factor that promotes fat deposition by regulating adipocyte differentiation and adipogenesis in buffalo.

## Introduction

Beef (cattle meat) is the third most widely consumed meat worldwide. It is a consumer favorite because of its “flavor,” “tenderness,” “juiciness,” and “rich in nutrition” properties (1). Buffalo meat has the same nutritional values as beef (2), but it is not widely accepted by consumers because of its low intramuscular fat (IMF) content which negatively affects its flavor, tenderness, and juiciness (3). Since China is the most populous country in the world, beef is often in short supply. Therefore, enhancing IMF deposition in buffalo meat has become one of the major goals in current buffalo breeding activity.

Adipogenesis is a well-orchestrated multistep process that involves the action of a large number of transcription factors (4–6), and in particular the PPAR (PPARγ, PPARα) (4, 7–10) and C/EBP family (C/EBPα, C/EBPβ, and C/EBPδ) (4, 9, 11) are enriched for adipogenesis regulated transcription factors in many animals species. Among them, PPARG (PPARγ) and C/EBPα, as key transcription factors in adipogenesis, are involved in a single pathway of adipocyte development with PPARγ being the proximal effector of adipogenesis (12). The master regulatory factors affecting adipogenesis have been widely studied, but it is far from enough to analyze the molecular regulatory mechanism of fat deposition only by transcription factors. Currently, several new factors are being proposed as regulators or influencers of adipogenesis. For example, sterol regulatory element binding protein (SREBP) transcription factor (13), phosphoenolpyruvate carboxykinase1 (*PCK1*) (3, 14), and fatty acid binding protein (*FABP4*) (9). In addition, non-coding RNAs (miRNAs, lncRNAs, and circRNAs) also play an important role in regulating the economic traits of livestock and poultry, and they interact with coding RNAs to form a regulatory network to jointly regulate fat deposition (15).

circRNAs, a type of endogenous non-coding RNA with covalently closed loop structure (16, 17), have become a research hotspot in recent years. They have been recently reported to be involved in multiple biological processes, such as cancer (18–20), ontogenesis (21, 22) and adipogenesis (23, 24). RNA sequencing technology has been instrumental to show that circRNAs modulate fat deposition in livestock animals (25–27). In pig, the potential lncRNAs/circRNAs-miRNAs-mRNAs regulatory networks shared *MYOD1, PPARD*, miR-423-5p and miR-874, which were associated with skeletal muscle muscular proliferation, differentiation/regeneration, and adipogenesis (28). In chicken, several reference circRNAs, such as circLCLAT1, circFNDC3AL, circCLEC19A, and circARMH1, potentially affect adipogenesis by regulating miRNAs via PPAR and fatty acid metabolism-related pathways (26). Recent studies have shown that circINSR inhibits preadipocyte adipogenesis in bovine by alleviating inhibition of miR-15/16 against target genes (29, 30). In this study, a candidate circRNA 21:6969877|69753491(circMARK3) was found through weighted gene co-expression network analysis (WGCNA), and further gain-of-function experiments demonstrated that circMARK3 promoted the adipogenic differentiation of buffalo adipocytes by up-regulating the adipogenesis relative gene. In summary, we propose a potential circRNA that plays an important role in buffalo fat deposition, which provides a molecular basis for beef quality improvement.

## Materials and Methods

### Animal Ethics

Six Chinese swamp buffaloes were bred for commercial use, rather than for experimental reasons, and they were slaughtered according to the food industry-approved halal food quality certified protocol by a Muslim cleric according to the law of Islam. Thus, no ethics approval was required by a specific committee (31).

### Animals and Tissue Samples

Six Chinese swamp buffaloes were raised at the Xinyang buffalo farm (Xinyang, Henan, China) with equivalent forage and feeding management conditions. Animals were weaned at 6 months of age and slaughtered at 30 months of age. Tissues, i.e., heart, liver, spleen, lung, kidney, longissimus dorsi muscle, and back subcutaneous fat, were sampled immediately after slaughter and were frozen in liquid nitrogen for RT-qPCR experiments. For primary adipocyte isolation, fresh back subcutaneous fat tissue was sampled, kept in phosphate buffer saline (PBS) with 1% streptomycin and penicillin, and taken back to the lab for isolation and culture of adipose tissue-derived mesenchymal stem cells.

### CircRNA Bioinformatics Analysis

The circRNAs expression matrix and DE circRNAs obtained from RNA-seq analysis have been reported previously (31). The WGCNA package in R4.1.0 provides a comprehensive set of functions for performing weighted correlation network analysis (32), and phenotypic information used in WGCNA is shown in Supplementary Table S1. The overlapping circRNAs among DE circRNAs and co-expression modules were analyzed using VENNY 2.1(https://bioinfogp.cnb.csic.es/tools/venny/index.html).

### Weighted Gene Co-expression Network Analysis

The co-expression network of the circRNAs was constructed base-on-base using the circRNAs expression matrix (33) and traits characteristic data (Supplementary Table S1). To allow direct comparison between sample, circRNAs in each sample were normalized as the number of back-spliced reads per million mapped reads (RPM) (31, 34, 35). The soft threshold for co-expression network construction was determined and the adjacency matrix was defined. The adjacency matrix was subsequently converted to a topological overlap matrix (TOM) and the corresponding dissimilarity TOM (dissTOM) was calculated. For modules with high TOM, the adaptive dynamic pruning algorithm was used to merge the modules. The soft-free network was constructed using the module function, after which module partition analysis was performed to identify the gene co-expression modules. Gene significance (GS) and module membership (MM) values were calculated. MM is the correlation coefficient between a gene and the genes for trait characteristics within the module and can be used to screen for important genes in the module. When GS and MM values of a gene in a module show significant correlation, it suggests that the gene may be a hub gene that is highly correlated with the target trait.

### RNA Isolation and cDNA Synthesis

Total RNA was isolated by TRIzol (Invitrogen, Carlsbad, CA, United States) according to the manufacturer's instructions. RNA quality was measured with NanoDrop 2000 (Nanodrop, Wilmington, DE, USA) and 1.5% agarose gels. RNA with 1.8 < 260/280 value < 2.0 was used for further analysis. Elimination of linear RNA was performed by Lucigen RNR07250 Ribonuclease R kit (RNase R, Lucigen-Simplifying Genomics). RNase R reaction system according to the manufacturer's instructions. Isolation of nuclear and cytoplasmic RNA was performed using the PARIS kit (Life Technologies, Carlsbad, CA, United States) according to the manufacturer's instructions. The total RNA was transcribed into cDNA using the PrimeScript^TM^ RT Master Mix (Takara, Dalian, China).

### RT-qPCR Analysis

Glyceraldehyde-3-phosphate dehydrogenase (*GAPDH*) and β*-actin* were used as reference genes. Furthermore, β*-actin* was used as a cytoplasmic marker in both nucleus and cytoplasm for cell localization. RT-qPCR was performed using TB Green^TM^ Premix Ex Taq^TM^ II (Dalian, China, Takara Bio) and LightCycler® 96 (Switzerland, Roche) with two-step reactions according to the manufacturer's recommended protocol. The 2^ΔΔCt^ method was used to calculate the relative expression level of circRNA. Three replicates were run per sample and the RT-qPCR experiment was performed three times. Among them, primers were designed using the “pick primers” function from NCBI (https://www.ncbi.nlm.nih.gov/tools/primer-blast/) and Primer 5.0 (Supplementary Table S2).

### Vector Construction and Adenovirus Packaging

For 3T3-L1 cells, mouse-circMARK3 (mouse-source sequence) overexpression was achieved by using pCD2.1-ciR vector. Mouse pCD2.1-circRNA was amplified from cDNA of mouse adipose tissue. The sequence of mouse circRNA (Supplementary Text S1) was cloned into the KpnI and BamHI restriction sites of the pCD2.1-ciR vector. However, due to the fact that buffalo primary adipocytes are difficult to efficiently transfect, the overexpression of buffalo-circMARK3 (buffalo-source sequence) was achieved by adenovirus packaging experiments (36). Adenovirus packaging was performed at Hanbio Biotechnology Co., Ltd. (Shanghai, China). Briefly, full length buffalo-circRNAs (Supplementary Text S1) were synthesized and ligated to the AdMax system to obtain Ad-circRNA. EGFP was used as an indicator for transduction efficiency and Ad-EGFP was used as a negative control.

### Cell Transfection, Adenovirus Transduction, Oil Red O Staining and Quantification

For 3T3-L1 preadipocytes, transfection with mouse pCD2.1-circRNA was performed using Lipofectamine 3000 (Invitrogen, Carlsbad, CA, United States) when cells reached 80% confluence, following the manufacturer's protocol. Forty-eight hours after transfection, 3T3-L1 primary adipocytes were treated for 2 days with inducing medium containing 1 μM dexamethasone (Sigma, USA), 0.5 mM IBMX, 10 μg/mL insulin and 1 μM rosiglitazone (Sigma, Milwaukee, WI, USA). Then, 3T3-L1 cells were treated with a maintenance medium containing 10 μg/mL insulin and 1 μM rosiglitazone. The maintenance medium was replaced every 2 days for a total of 6 days, until induction of differentiation. Similar to cell transfection, adenovirus transfection buffalo Ad-circRNA was performed when buffalo adipocytes reached 80% confluence according to the adenovirus transduction manufacturer's protocol. Two days after transfection, buffalo adipose-derived mesenchymal stem cells were treated with inducing medium for 2 days. For a further 4 days, they were treated with maintenance medium, which was changed every 2 days. After inducing with adipogenic agents for 6 days, Oil Red O staining and quantification were performed as described (36).

### Statistical Analysis

Comparisons were analyzed using SPSS software. *P*-value < 0.05 was considered to indicate statistical significance. Results are represented as the mean ± SD (*n* = 3) and plotted with GraphPad Prism7 software.

## Results

### Weighted Gene Co-expression Network Analysis

**Figure 1:**
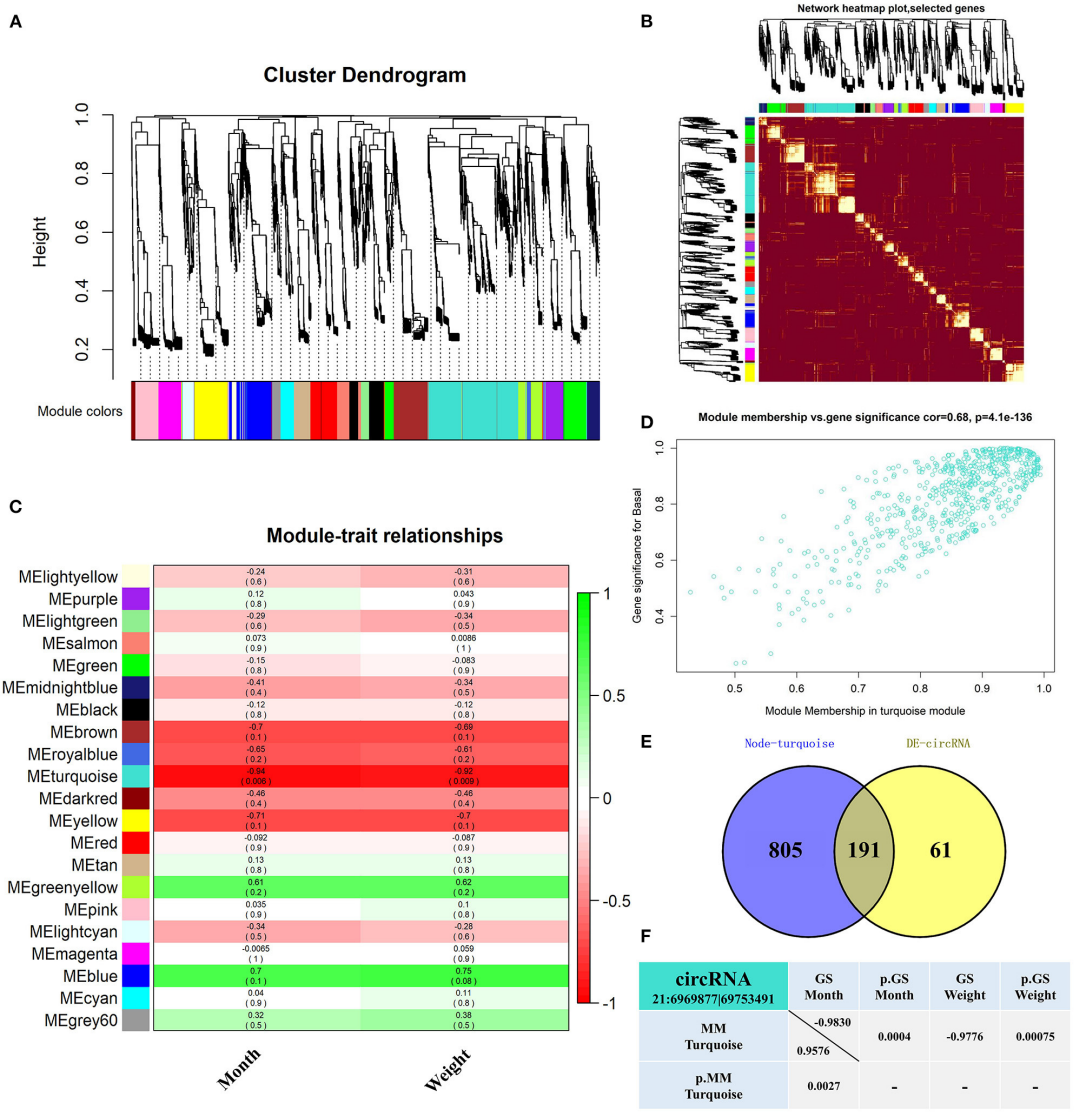
Screened-out circRNAs significantly associated to buffalo fat deposition. **(A–D)** CircRNA co-expression network of adipose tissue in the Xinyang buffalo revealed by WGCNA; **(A)** Hierarchical cluster tree of co-expression modules, where every leaf on a tree is a circRNA and main branches are made up of 21 color-coded modules; **(B)** Construction of co-expression modules by WGCNA. Progressively more saturated red colors indicate higher overlap among these functional modules and blocks of lighter color along the diagonal are the gene modules. Module assignment gene and dendrograms are at the top and left, respectively; **(C)** Association analysis of gene co-expression network modules with trait, where each row corresponds to a module (name displayed on the left) and each column corresponds to a particular trait. Colors of the row/column intersection cells indicate the correlation coefficient between module and trait (positive-green and negative-red); **(D)** Scatterplot of Gene Significance (GS) For Traits vs. Module Membership (MM) in the turquoise module, with a highly significant correlation between GS and MM (one dot represents one gene in the turquoise module); **(E)** Overlapping circRNAs between DE circRNAs and co-expression turquoise module; **(F)** CircRNA 21:6969877|69753491 GS and MM values.

WGCNA analyzed 5,141 circRNAs obtained from RNA-seq (31) and was used to construct 21 co-expression modules (Figure 1A). Among them, the soft-thresholding power we chose was 9 as the correlation coefficient threshold, and 30 was chosen as the minimum number of circRNA in modules. To merge possible similar modules, we defined 0.25 as the threshold for cut height. The modules comprising most genes were turquoise, followed by blue, brown, and yellow (Supplementary Table S3). Moreover, the dissTOM obtained was subjected to hierarchical clustering, resulting in a hierarchical clustering tree (5,000 circRNAs), and these modules were independent of other modules (Figure 1B). Module-trait correlation analysis showed that the turquoise module was related to month and weight (Figure 1C). The significance of these circRNAs in the turquoise module is shown in Figure 1D and Supplementary Table S4. We found 191 overlapping circRNAs between the DE circRNAs list (31) and the turquoise module (Supplementary Table S5; Figure 1E). Among these, circRNA 21:6969877|69753491 has been shown to be involved in lipid metabolism in a previous study, therefore it likely affects fat deposition. Gene significance (GS>0.8) (37, 38) and module membership (MM>0.8) (37–39) values showed a high correlation between circRNA 21:6969877|69753491 and two traits (month and weight) of buffalo Figure 1F.

### Characteristic Analysis of CircMARK3

**Figure 2:**
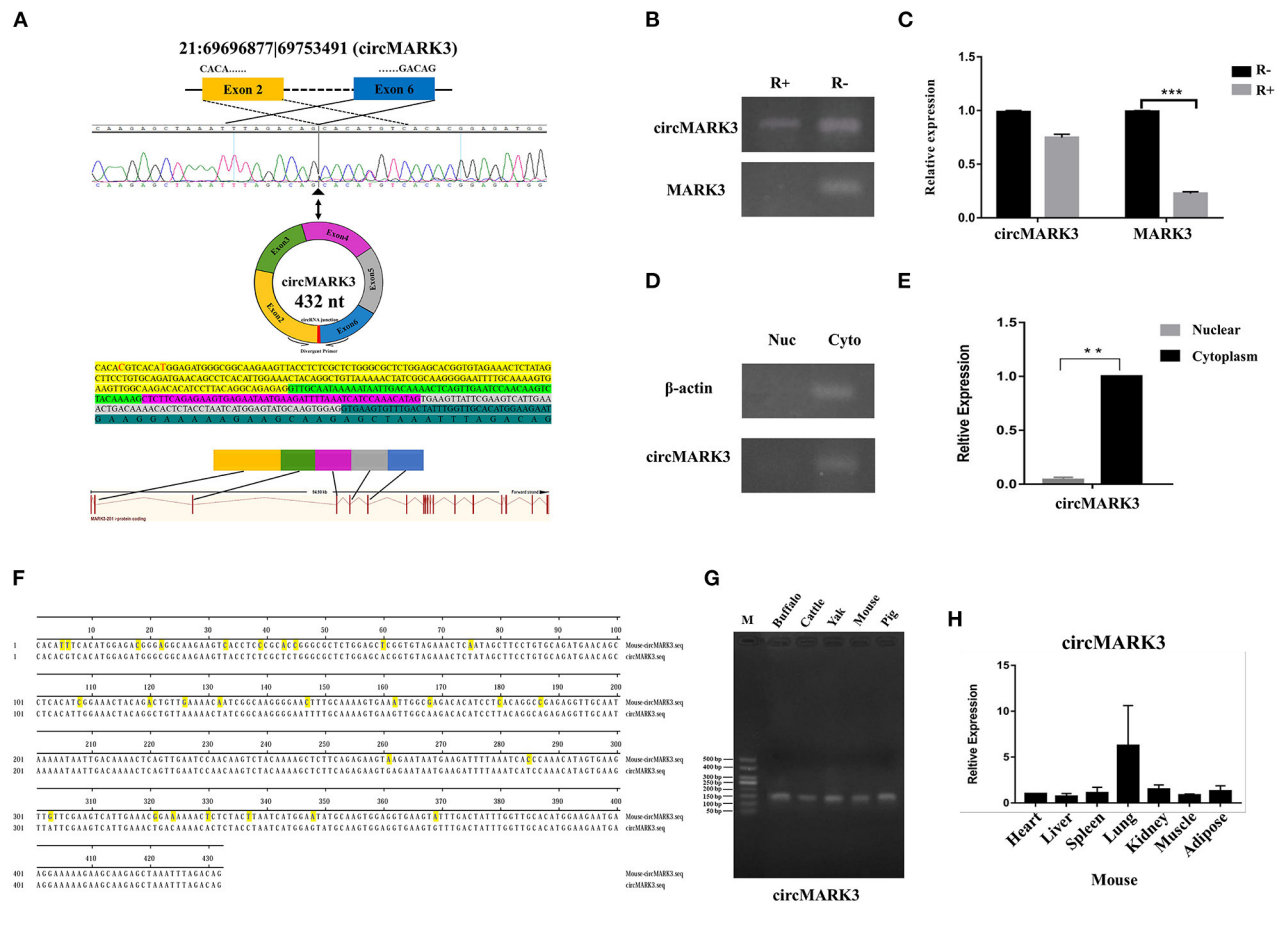
Characteristic analysis of circMARK3. **(A)** Basic information of circMARK3 composition structure (bases C and T labeled red were mutated into T and C in the sequence); **(B)** Semiquantitative PCR results of circMARK3 and *MARK*3 before and after RNase R treatment; **(C)** RT-qPCR results of circMARK3 and *MARK*3 before and after RNase R treatment; **(D)** Cell localization of circMARK3 by semiquantitative PCR; **(E)** Cell localization of circMARK3 by RT-qPCR; **(F)** Sequence alignment results of circMARK3 in mouse and buffalo; **(G)** CircMARK3 conservation in adipose tissues of different species by semiquantitative PCR detection; **(H)** Expression profile of circMARK3 in heart, liver, spleen, lung, kidney, muscle and adipose tissues of mouse. In panels B-E, bovine-β*-actin* was used to normalize the expression level of the reference gene. In panel H, mouse-β*-actin* was used to normalize the expression level of the reference gene. Data are presented as the mean ± SD, *n* = 3, ^**^P < 0.01, ^***^P < 0.001. **(B–D)** The samples derive from the same experiment and gels were processed in parallel.

Since circRNA 21:6969877|69753491 (432 nt) is formed by splicing and circularizing exons 2–6 of the microtubule affinity regulating kinase 3 (*MARK*3) gene (Figure 2A), we have named it circMARK3. Semiquantitative PCR results show the latter still exists in primary adipocytes by the RNase R digestion linear RNA (Figure 2B). The RT-qPCR results show that circMARK3 is still highly expressed in cells treated by the RNase R (Figure 2C). These results show that circMARK3 is stably expressed as circRNA (Figures 2B,C). Also, semiquantitative PCR and RT-qPCR results of isolated nuclear and cytoplasm of adipocytes show that circMARK3 is mainly expressed in the cytoplasm (Figures 2D,E). Conservation in different species was assessed by semiquantitative PCR and RT-qPCR detection: circMAK3 is conserved in buffalo, cattle, yak, mouse and pig (Figure 2G), and is ubiquitously expressed in different tissues of mice (Figure 2H). Sequence alignment shows that circMARK3 in mouse and buffalo are partially conserved, and the homology was 93.51% (Figure 2F).

### The Expression Pattern of CircMARK3

**Figure 3:**
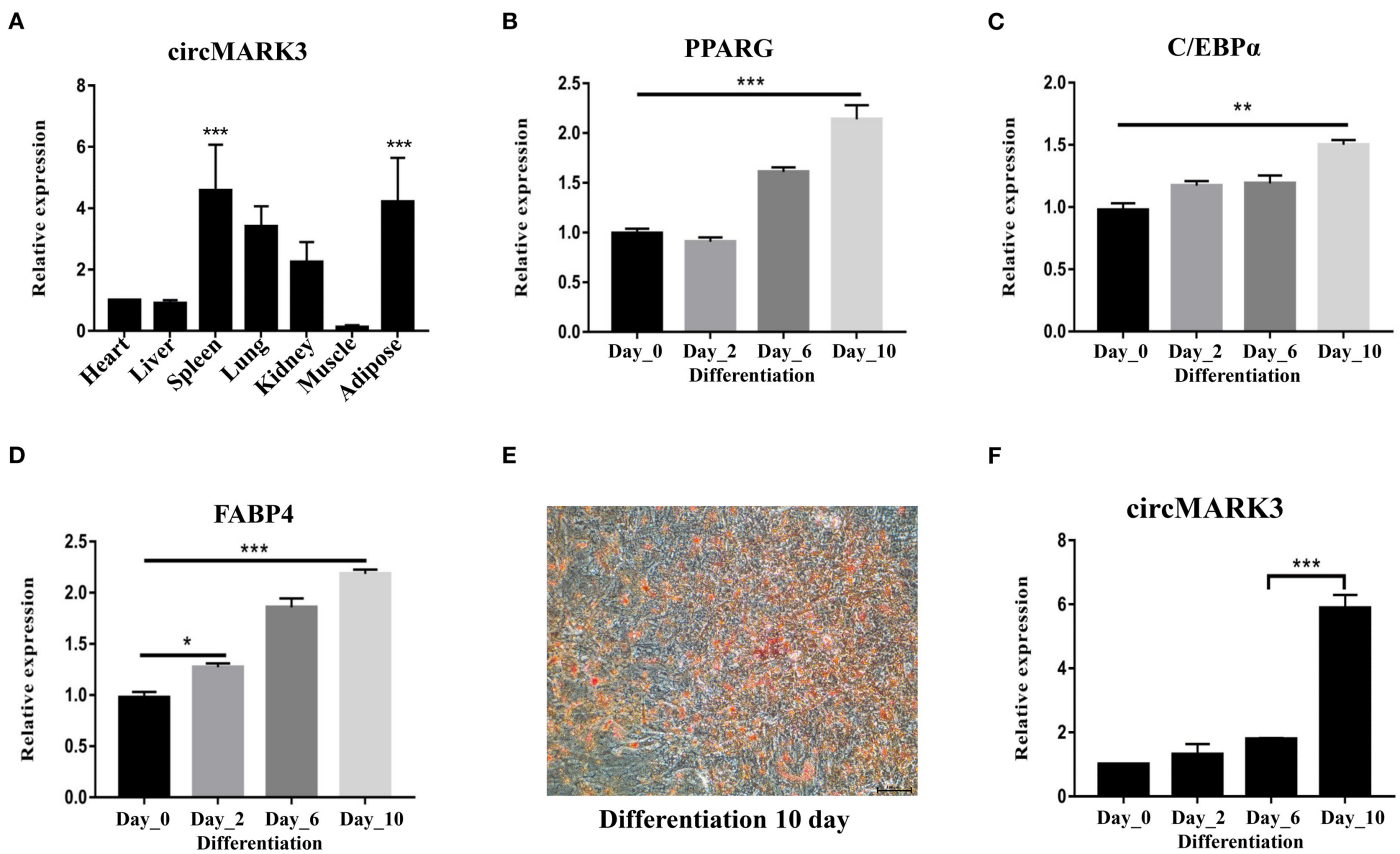
Expression pattern of circMARK3 in tissues and adipocytes of buffalo. **(A)** Expression profile of circMARK3 in heart, liver, spleen, lung, kidney, muscle and adipose tissues of buffalo; **(B–D)** expression pattern during buffalo adipocyte differentiation of *PPARG* (after 0, 2, 6, and 10 days) **(B)**, *C/EBP*α **(C)** and *FABP*4 **(D)**; **(E)** Oil Red O staining for detection of buffalo adipocyte lipid deposition (differentiation day 10); **(F)** Expression pattern of circMARK3 during buffalo adipocyte differentiation. In **(A–D)** and **(F)**, bovine-*GAPDH* was used to normalize the expression level of the reference gene. In panel **(A)**, data was normalized to the heart. In panels **(B–D)** and **(F)**, all data were normalized to day 0. Data are presented as the mean ± SD, *n* = 3, #:no significance, ^*^P < 0.05, ^**^P < 0.01, ^***^P < 0.001.

circMARK3 is mainly expressed in adipose tissue of buffalo (Figure 3A, *p* < 0.001). Primary adipocytes cultured *in vitro* are ideal cell models for studying the molecular regulatory mechanism of adipogenesis. Therefore, buffalo adipocytes were successfully isolated and cultured (Figures 3B–D). By inducing the culture medium, they successfully differentiated to produce lipid droplets (Figure 3E). During adipogenic differentiation, circMARK3 was up-regulated in the mature adipocytes (Figure 3F).

### CircMARK3 Promotes the Adipogenic Differentiation of 3T3-L1 Cells

**Figure 4:**
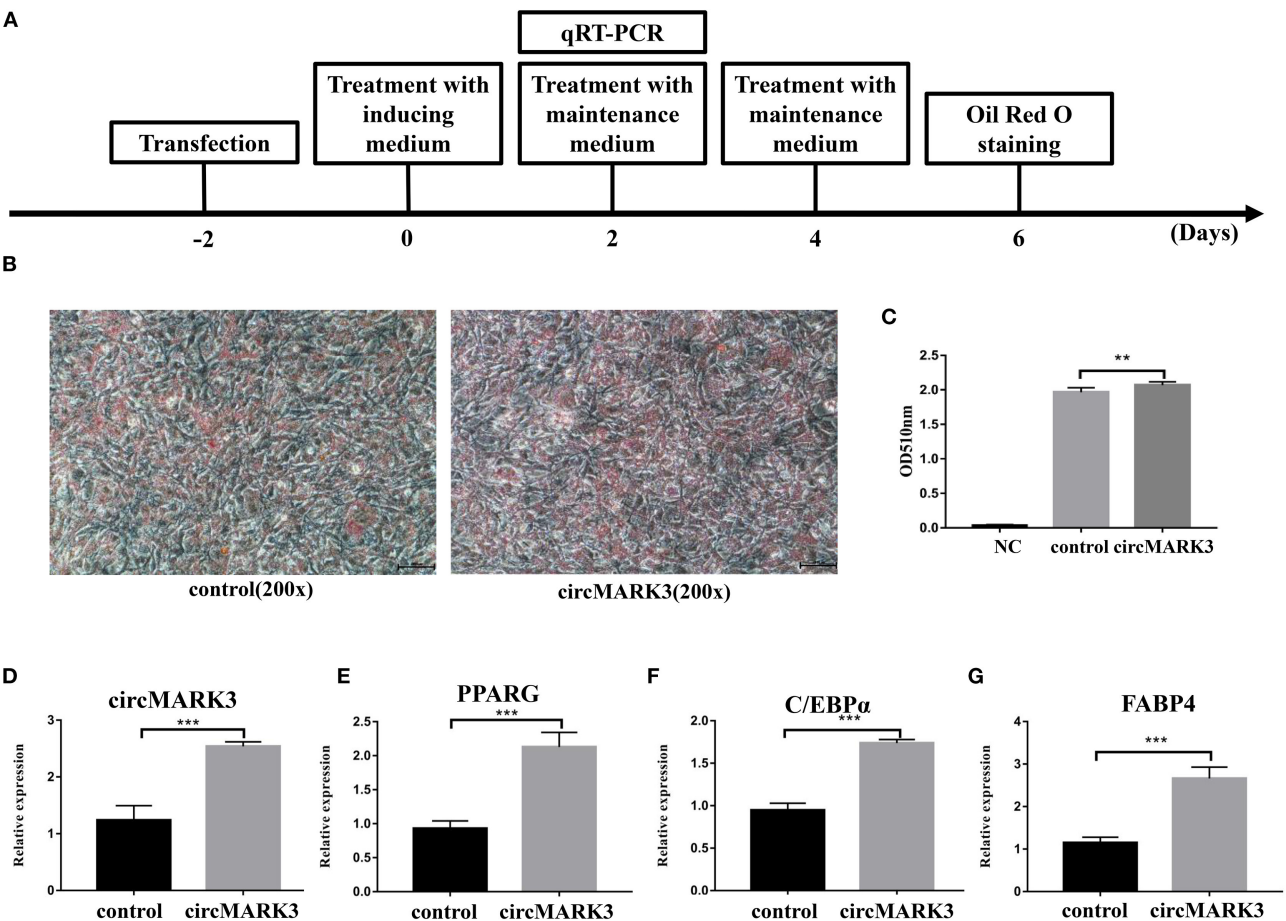
Overexpression of mouse circMARK3 promotes adipogenic differentiation of 3T3-L1 adipocytes. **(A)** Strategy for mouse circMARK3 overexpression, adipogenic differentiation, RT-qPCR and Oil Red O staining in 3T3-L1 cells; **(B)** Oil Red O staining in 3T3-L1 cells transfected with pCD2.1-ciR and mouse pCD2.1-circMARK3 on day 6 after adipogenic differentiation. **(C)** Histogram corresponding to the quantification of Oil Red O staining by spectrophotometry; **(D–G)** RNA expression 48 h after transfection of circMARK3 **(D)**, *PPARG* **(E)**, *C/EBP*α **(F)** and *FABP*4 **(G)**. In panels **(D–G)**, mouse-β*-actin* was used to normalize the expression level of the reference gene in 3T3-L1 cells. Control means the pCD2.1-ciR group, circMARK3 means the mouse pCD2.1-circMARK3 group. NC means negative control. Data are presented as the mean ± SD, *n* = 3, ^**^P < 0.01, ^***^P < 0.001.

The sequences of mouse and buffalo circMARK3 show high homology (Figures 2F,H). Therefore, the role of circMARK3 in fat deposition was investigated by performing gain-of-function experiments for mouse-circMARK3 in 3T3-L1 cells. The strategy of transfection, adipogenic differentiation, RT-qPCR and Oil Red O staining is shown in Figure 4A. Consistent with a higher lipid accumulation in the mouse pCD2.1-circMARK3 group than in the pCD2.1-ciR group (Figures 4B,C, *P* < 0.01), the former group showed much higher circMARK3 mRNA expression (Figure 4D, *P* < 0.001) and significant higher up-regulation of *PPARG, C/EBP*α, and *FABP*4 (Figures 4E–G, *P* < 0.001).

### CircMARK3 Promotes the Adipogenic Differentiation of Buffalo Adipocytes

**Figure 5:**
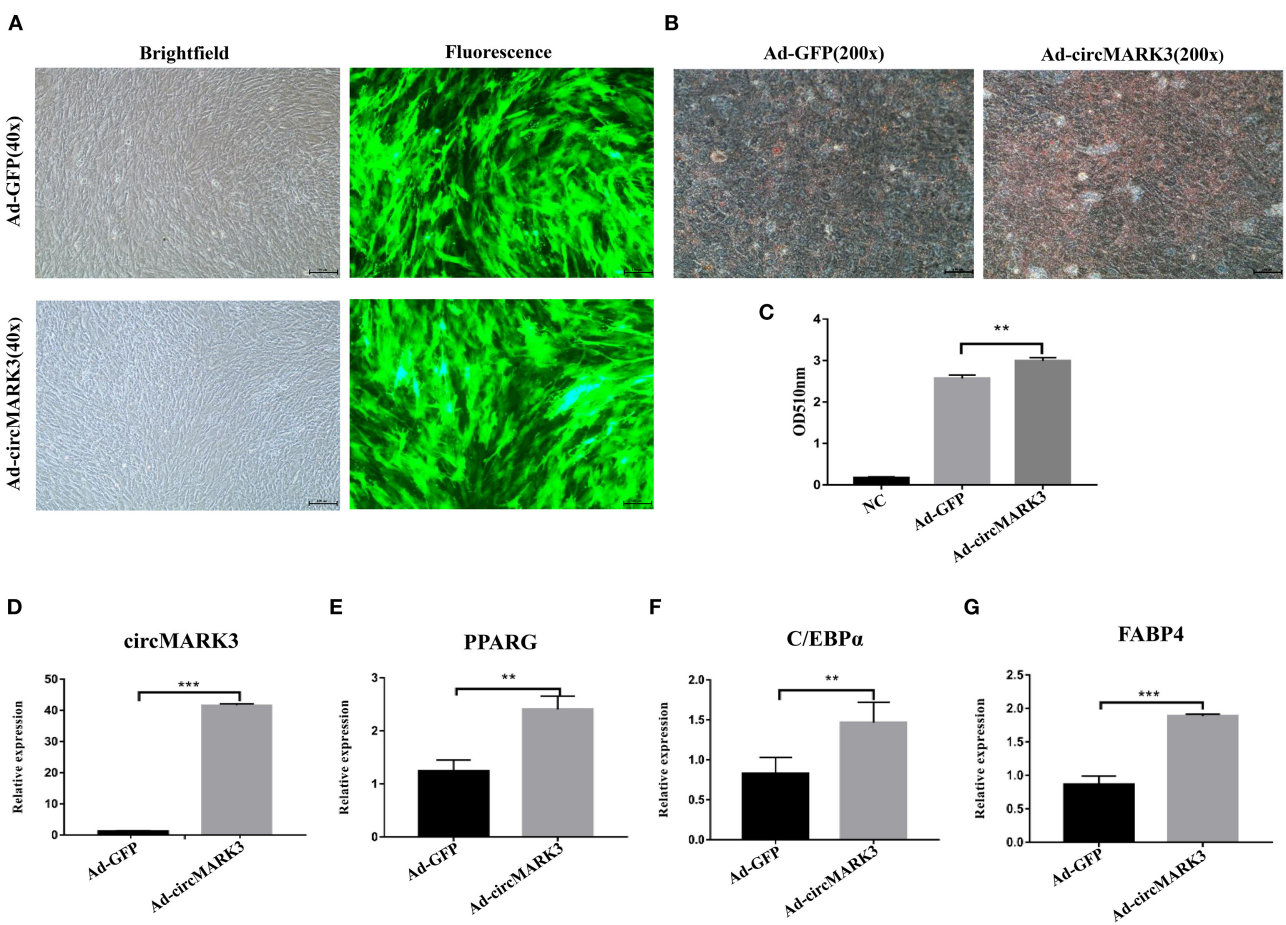
Overexpression of buffalo circMARK3 promotes adipogenic differentiation of buffalo adipocytes. **(A)** Micrographs of GFP-positive cells in the Ad_GFP and Ad_circMARK3 groups on day 2 after cell transfection; **(B)** Oil Red O staining of buffalo adipocytes transfected with Ad_GFP and Ad_circMARK3 group on day 6 after adipogenic differentiation. **(C)** Histogram corresponding to the quantification of Oil Red O staining by spectrophotometry; **(D–G)** RNA expression two days after transfection of circMARK3 **(D)**, *PPARG* **(E)**, *C/EBP*α **(F)**, and *FABP*4 **(G)**. Bovine-*GAPDH* was used to normalize the expression level of the reference gene in buffalo adipocytes. NC means negative control. Data are presented as the mean ± SD, *n* = 3, ^**^P < 0.01, ^***^P < 0.001.

To evaluate the effect of circMARK3 on fat deposition in buffalo, full length buffalo-circMARK3 was packaged into an adenovirus system for overexpression (Ad_circMARK3), following the same scheme shown in Figure 4A. The indicator GFP was highly expressed 2 days after adenoviral transduction (Figure 5A). Expression of circMARK3 in Ad_circMARK3 was significantly higher than in the Ad_EGFP group on day 2 of cell transfection (Figure 5D, *P* < 0.001). At the same time, lipid accumulation in Ad_circMARK3 was significantly enhanced (Figures 5B,C, *P* < 0.01), whereas mRNA expression of *PPARG, C/EBP*α, and *FABP*4 was slightly up-regulated on day 2 after transfection (Figures 5E–G, *P* < 0.01).

## Discussion

Fat deposition is closely related to growth and development (40, 41), and IMF is a key factor affecting beef quality (42). Compared to beef, however, the IMF content in buffalo meat is significantly lower (3). Since buffalo is abundant in China, it would be desirable to increase its IMF content. It is known that the IMF correlates with maturity (43) and percentage (44) of back subcutaneous fat, which therefore may be used as IMF indicator. Therefore, determining the related factors affecting subcutaneous fat deposition can provide theoretical basis for improving meat quality.

WGCNA is an efficient and accurate method based on RNA-seq used for biological data mining (45, 46). This method is increasingly used to discover genes and phenotype relationships (46, 47) and to provide insights into signaling networks linked to phenotypic traits of interest (46, 48). From the circRNAs obtained by RNA-seq, the top 5,000 with median absolute deviation (MAD) were used to construct the network for WGCNA, resulting in 21 modules. When the scale-free topology index was 0.9, the resulting network was closer to a power law distribution (46), but the appropriate soft threshold was not found (Supplementary Figures S1A,B). This may be caused by differences between samples, but they are caused by meaningful biological changes. Here, we used experience soft threshold for analysis. We found that the blue and turquoise modules were positively and negatively correlated with traits, respectively. However, since the blue module had no biological significance (*P* > 0.05), the turquoise module, containing 996 circRNAs, was the main one involved in traits of buffalo (month and weight). Taking the intersection between DE circRNAs and the turquoise modules (49), circRNA 21:6969877|69753491 was taken as a candidate factor affecting fat deposition.

The activities in living organisms are mediated by the genome (50), and gene expression is regulated by many factors. Adipocytes may include white adipocytes that store energy and brown or beige adipocytes that dissipate energy (51, 52). Transcription factor PRDM16 is a beige/brown marker (53) associated with circRNA 21:6969877|69753491 by RNA-seq analysis (31). In addition, knockout mice (MARK3^−/−^) were protected against high-fat diet induced obesity and displayed attenuated weight gain (54). Both WGCNA and RNA-seq analysis indicate that circRNA 21:6969877|69753491 produced by *MARK*3 gene are likely involved in fat deposition. By digestion linear RNA experiments detecting the expression of the circRNA and host genes, the expression of host genes was significantly down-regulated. Although expression of circRNA was too reduced, it was still detected. circMARK3 was highly and stably expressed in buffalo adipocytes, it may be a candidate gene for influencing fat deposition in Buffalo. The function of circRNAs is determined by location (55–58), which therefore must be determined: in the nucleus, it usually participates in regulating expression of host genes (55, 56), whereas in the cytoplasm, it mainly acts as competitive endogenous RNA (ceRNA) (57, 58). Since we found that circMARK3 is mainly expressed in the cytoplasm, it may function as ceRNA. Localization is useful for future exploration of its molecular regulatory mechanism of adipose tissue development.

Since the expression patterns showed that circMARK3 is mainly expressed in adipose tissue, we speculated that it plays an important role in buffalo adipogenesis. To explore its function, we obtained buffalo adipocytes and detected circMARK3 expression during the different phases of differentiation. Adipocytes were isolated from buffalo back subcutaneous adipose tissue (36, 59) and induction of differentiation was followed by staining with Oil Red O solution. The results showed that there was a significant increase in intracellular lipid droplets after induction, which indicated that the primary adipocytes had strong differentiation activity (4). Expression of marker genes *PPARG, C/EBP*α, and *FABP*4 increased during adipocyte differentiation, reaching its highest level at day 10, consistent with previous studies (60, 61). These results suggested the establishment of a differentiation induction system for buffalo primary adipocytes. Adipocytes were active and could be used in subsequent experiments. During adipogenic differentiation, the expression of circMARK3 was up-regulated in the mature adipocytes of buffalo, suggesting its involvement in adipocyte differentiation.

The host gene *MARK*3 has been linked to lipid metabolism in mice (54), and we have shown that circMAK3 is conserved in mice. To confirm the effect of circMARK3 on fat deposition in mice and buffalo, overexpression of this circRNA in 3T3-L1 cells was performed using a pCD2.1-ciR overexpression vector. In buffalo adipocytes, overexpression was performed by an efficient adenovirus system. pCD-ciR is a more commonly used circRNAs expression vector. The linear sequence of the target circRNAs was amplified by PCR and cloned into the pCD-ciR vector, which contains the circRNAs circular expression framework. After the recombinant vector is transfected into the cells, the RNA can be sheared to form circRNAs molecules with high efficiency and stability to achieve high expression in cells. As expected, circRNAs not only significantly enhanced adipogenic differentiation of 3T3-L1, but also the accumulation of lipid droplets in buffalo adipocytes. In both systems, the expression of adipogenic marker genes *PPARG, C/EBP*α and *FABP*4 positively correlated with the degree of the differentiation of adipocytes (4, 62) was up-regulated. These results suggest that circMARK3 promotes adipogenesis by enhancing the expression of adipogenic marker genes, but the regulatory mechanism involved in enhancing adipogenic differentiation in 3T3-L1 and buffalo adipocytes requires additional investigation.

## Conclusions

**Figure 6:**
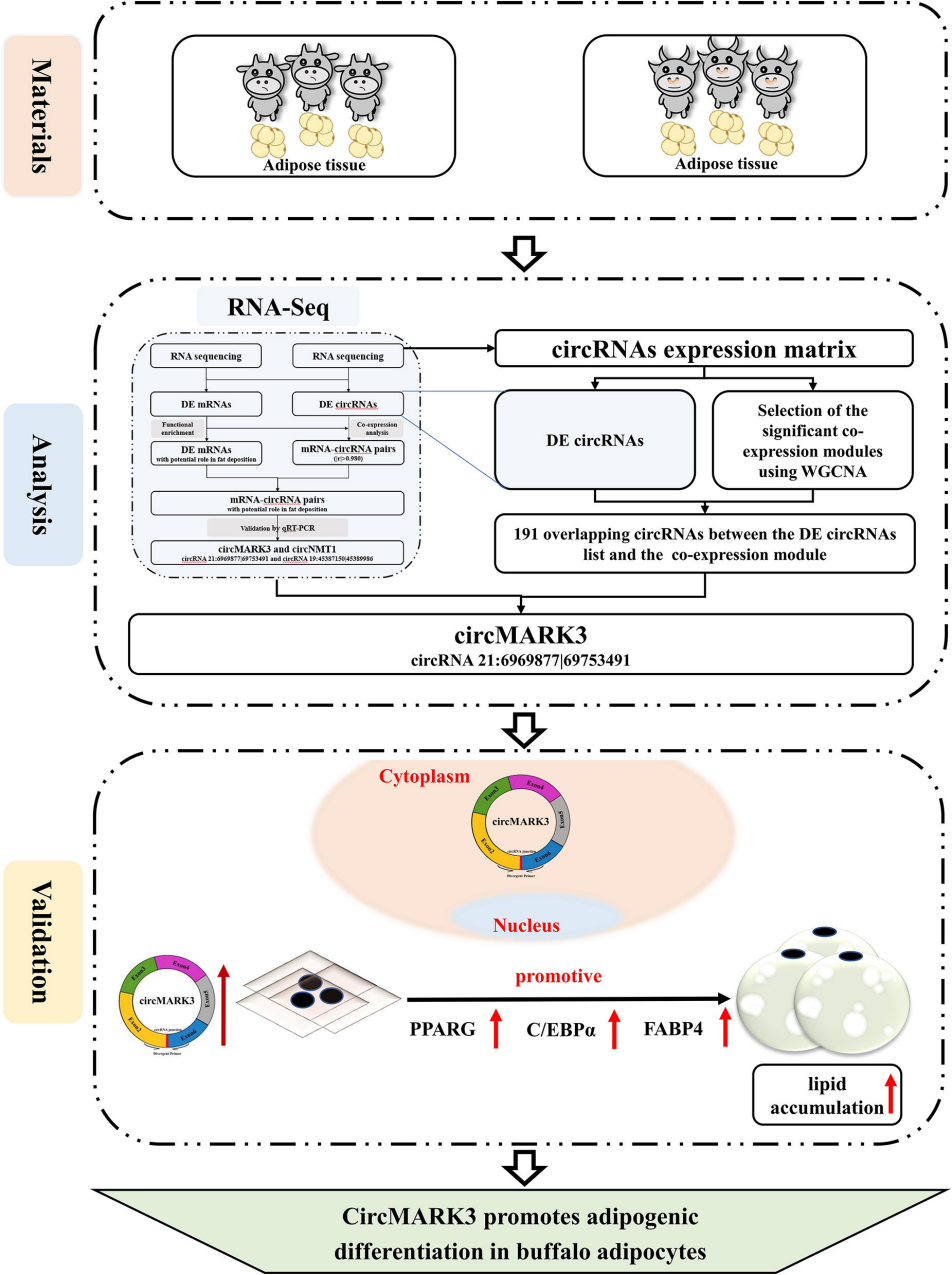
Schematic illustration of the experimental procedure and main results. RNA-seq analysis is from a previous study (31).

The central idea and results of this research are illustrated in Figure 6. This study demonstrates that: (1) A candidate circRNA circMARK3 related to lipid metabolism was found by WGCNA, (2) circMARK3 is highly expressed in adipose tissue and mature adipocytes and is located in the cytoplasm, (3) circMARK3 promoted adipogenic differentiation of buffalo adipocytes and 3T3-L1 cells by up-regulating the expression levels of adipogenic marker genes *PPARG, C/EBP*α and *FABP4*. All in all, the study suggests that circMARK3 is a potential regulatory factor affects buffalo fat deposition, but the regulatory mechanism involved in fat production needs further exploration.

## Data Availability Statement

The original contributions presented in the study are included in the article/Supplementary Material, further inquiries can be directed to the corresponding author.

## Ethics Statement

Ethical review and approval was not required for the animal study because all animals were bred for commercial use, rather than for experimental reasons, and they were slaughtered according to the food industry-approved halal food quality certified protocol by a Muslim cleric according to the law of Islam. Thus, no ethics approval was required by a specific committee.

## Author Contributions

Conceived and designed the research: YM and FL. Provided the funding: YM. Analyzed the data and wrote the paper: XF. Conducted the experiment: JZ. Modified manuscript: FL, BA, and AA. All authors contributed to the article and approved the submitted version.

## Funding

This study was funded by the National Natural Science Foundation of China (32072720), The Leading Talents Fund in Science and Technology Innovation in Henan Province (No. 194200510022), Ningxia Hui Autonomous Region Key Research and Development Project (2019YCZX0068, 2021BEF01002, and 2021NXZD1), and the Leading Talents Fund in Science and Technology Innovation in Ningxia Hui Autonomous Region (2020GKLRLX02).

## Conflict of Interest

The authors declare that the research was conducted in the absence of any commercial or financial relationships that could be construed as a potential conflict of interest.

## Publisher's Note

All claims expressed in this article are solely those of the authors and do not necessarily represent those of their affiliated organizations, or those of the publisher, the editors and the reviewers. Any product that may be evaluated in this article, or claim that may be made by its manufacturer, is not guaranteed or endorsed by the publisher.

## Abbreviations

WGCNA: Weighted gene co-expression network analysis
DE: Differentially expressed
MARK3: microtubule affinity regulating kinase 3
PPARγ or PPARG: peroxisome proliferator activated receptor gamma
C/EBPα: CCAAT/enhancer-binding protein α
FABP4: fatty acid binding protein 4
IMF: intramuscular fat
SREBP1: sterol regulatory element binding protein
THRSP: thyroid hormone responsive
PRDM16: PR/SET domain 16
PCK1: phosphoenolpyruvate carboxykinase 1
MYOD1: Myogenic Differentiation 1
PBS: phosphate buffer saline
GAPDH: Glyceraldehyde-3-phosphate dehydrogenase
SD: Standard Deviation
MAD: median absolute deviation
ceRNA: endogenous RNA
SE: standard error.
