# Supplementary material for: Weighted Gene Co-expression Network Analysis Revealed That CircMARK3 Is a Potential CircRNA Affects Fat Deposition in Buffalo
Source: Front Vet Sci. 2022 Jul 7;9:946447. doi: 10.3389/fvets.2022.946447 (PMC9302235; doi:10.3389/fvets.2022.946447)
Supplement: Supplementary file 6 [file Table_6.DOCX]

**Text S1** Information of mouse-circMARK3 and circMARK3 (buffalo-circMARK3) sequences

**Mouse-circMARK3 sequence**

CACATTTCACATGGAGACGGGAGGCAAGAAGTCACCTCCCGCACCGGGCGCTCTGGAGCTCGGTGTAGAAACTCAATAGCTTCCTGTGCAGATGAACAGCCTCACATCGGAAACTACAGACTGTTGAAAACAATCGGCAAGGGGAACTTTGCAAAAGTGAAATTGGCGAGACACATCCTCACAGGCCGAGAGGTTGCAATAAAAATAATTGACAAAACTCAGTTGAATCCAACAAGTCTACAAAAGCTCTTCAGAGAAGTAAGAATAATGAAGATTTTAAATCACCCAAACATAGTGAAGTTGTTCGAAGTCATTGAAACGGAAAAAACTCTCTACTTAATCATGGAATATGCAAGTGGAGGTGAAGTATTTGACTATTTGGTTGCACATGGAAGAATGAAGGAAAAAGAAGCAAGAGCTAAATTTAGACAG

**circMARK3 (Buffalo-circMARK3) sequence**

CACACGTCACATGGAGATGGGCGGCAAGAAGTTACCTCTCGCTCTGGGCGCTCTGGAGCACGGTGTAGAAACTCTATAGCTTCCTGTGCAGATGAACAGCCTCACATTGGAAACTACAGGCTGTTAAAAACTATCGGCAAGGGGAATTTTGCAAAAGTGAAGTTGGCAAGACACATCCTTACAGGCAGAGAGGTTGCAATAAAAATAATTGACAAAACTCAGTTGAATCCAACAAGTCTACAAAAGCTCTTCAGAGAAGTGAGAATAATGAAGATTTTAAATCATCCAAACATAGTGAAGTTATTCGAAGTCATTGAAACTGACAAAACACTCTACCTAATCATGGAGTATGCAAGTGGAGGTGAAGTGTTTGACTATTTGGTTGCACATGGAAGAATGAAGGAAAAAGAAGCAAGAGCTAAATTTAGACAG
