# Supplementary material for: Weighted Gene Co-expression Network Analysis Revealed That CircMARK3 Is a Potential CircRNA Affects Fat Deposition in Buffalo
Source: Front Vet Sci. 2022 Jul 7;9:946447. doi: 10.3389/fvets.2022.946447 (PMC9302235; doi:10.3389/fvets.2022.946447)
Supplement: Supplementary file 7 [file Table_7.pdf]

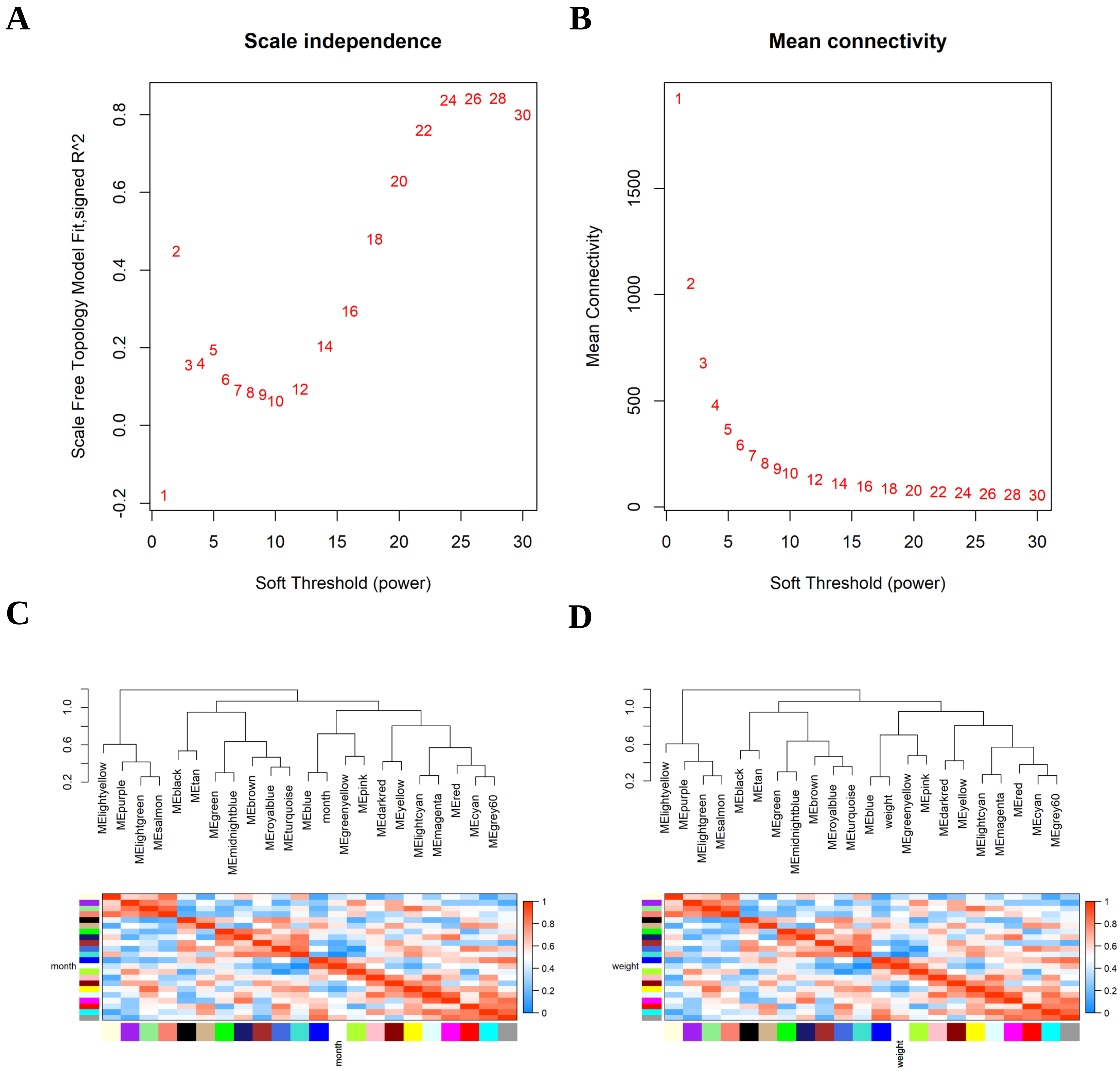

Figure S1. Analysis of network topology for various soft threshold and heat map of eigengene adjacency. (A) Analysis of the scale-free fit index for various soft threshold; (B) Analysis of the mean connectivity for various soft thresholds; (C-D) hierarchical clustering dendrogram of MEs (marked with corresponding colors) and traits. In the heatmap, red indicates high adjacency (positive correlation) and blue indicates low adjacency (negative correlation). The dendrograms and module gene are on the top, left and under, respectively.
